# Barriers and opportunities for gemcitabine in pancreatic cancer therapy

**DOI:** 10.1152/ajpcell.00331.2022

**Published:** 2022-12-26

**Authors:** Alica K. Beutel, Christopher J. Halbrook

**Affiliations:** ^1^Department of Molecular Biology and Biochemistry, University of California, Irvine, California; ^2^Department of Internal Medicine, University Hospital Ulm, Ulm, Germany; ^3^Chao Family Comprehensive Cancer Center, Orange, California

**Keywords:** chemoresistance, gemcitabine, metabolism, pancreatic cancer, tumor microenvironment

## Abstract

Pancreatic ductal adenocarcinoma (PDA) has become one of the leading causes of cancer-related deaths across the world. A lack of durable responses to standard-of-care chemotherapies renders its treatment particularly challenging and largely contributes to the devastating outcome. Gemcitabine, a pyrimidine antimetabolite, is a cornerstone in PDA treatment. Given the importance of gemcitabine in PDA therapy, extensive efforts are focusing on exploring mechanisms by which cancer cells evade gemcitabine cytotoxicity, but strategies to overcome them have not been translated into patient care. Here, we will introduce the standard treatment paradigm for patients with PDA, highlight mechanisms of gemcitabine action, elucidate gemcitabine resistance mechanisms, and discuss promising strategies to circumvent them.

## Graphical abstract

**Figure:**
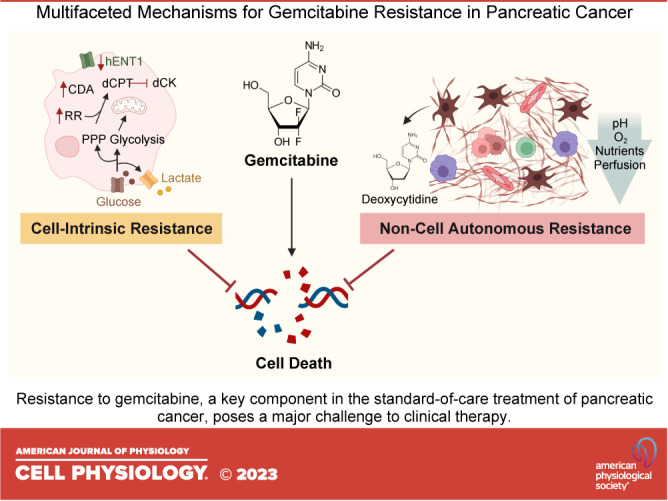

## INTRODUCTION

Pancreatic ductal adenocarcinoma (PDA) is one of the most lethal malignancies with a devastating 5-year survival rate of ∼11% for all stages combined (1). The poor prognosis is usually attributed to late diagnosis due to a lack of early specific symptoms combined with an exceptionally aggressive tumor biology. Resistance to chemo- and radiotherapy, targeted agents, and immunotherapy further complicates the treatment of PDA. Therefore, strategies to overcome resistance in PDA therapy are urgently needed to improve survival.

In this review, we will provide a brief overview of the standard-of-care treatments in PDA and highlight mechanisms of action for the frequently used nucleoside analog gemcitabine. We will discuss the multifaceted resistance mechanisms by which cancer cells are reprogrammed to overcome gemcitabine cytotoxicity, as well as the role of the tumor microenvironment (TME) in supporting chemoresistance. Finally, we will discuss current strategies that aim at overcoming resistance as an attempt to resensitize cancer cells to chemotherapy.

## CURRENT TREATMENT OPTIONS FOR PATIENTS WITH PDA

The pyrimidine antimetabolites gemcitabine (2) and 5-fluorouracil (3) were introduced as the first effective drugs for PDA in the late 1990s. Over the following decades, the therapeutic spectrum has expanded toward multiagent regimens based on these antimetabolite backbones. However, despite the evolution of chemotherapy modalities over the past decades, drug resistance remains a major hurdle.

**Figure 1.**
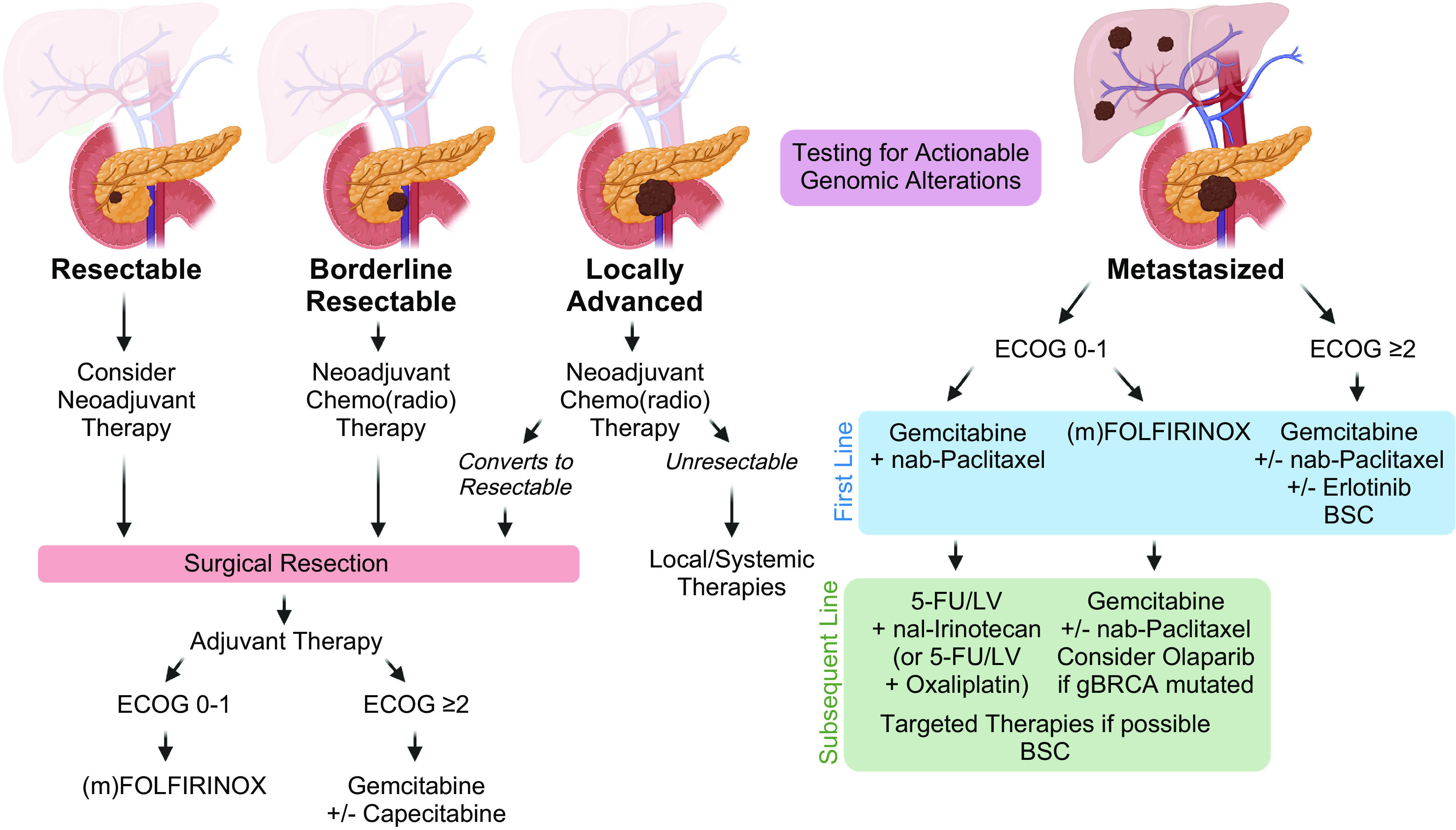
Treatment paradigm in pancreatic cancer. Recommended treatment approach for patients with PDA based on cancer stage. Patients are preferentially evaluated in a multidisciplinary tumor conference and clinical trials are encouraged whenever possible. Early testing for actionable genomic alterations is recommended for patients with advanced PDA. BSC, best supportive care; ECOG, Eastern Cooperative Oncology Group; 5-FU, 5-fluorouracil; Gem, gemcitabine; LV, leucovorin; (m)FOLFIRINOX, modified FOLFIRINOX (5-fluorouracil, irinotecan, leucovorin, oxaliplatin); nab, nanoparticle albumin-bound; nal, nanoliposomal; PDA, pancreatic adenocarcinoma. Image created with BioRender and published with permission.

Surgery is the preferred treatment approach for PDA; however, most patients are not eligible for resection at diagnosis. To assess resectability, the extent of tumor spread and encasement of critical vasculature are assessed by imaging, and tumors are classified as resectable, borderline resectable, locally advanced, or metastatic (4). Treatment recommendations are primarily based on this classification and are discussed in more detail in Fig. 1 and below.

### Resectable Tumors

Surgical resection, ideally combined with (neo)adjuvant therapy, provides the best survival outcome. However, only 15%–20% of patients meet the imaging criteria for resectability at the time of diagnosis. Although definitions are variable, the National Comprehensive Cancer Network (NCCN) defines a tumor with no arterial or venous tumor contact or ≤180° of contact to large veins as resectable (5). Apart from the anatomical localization, preoperative levels of the serum biomarker CA 19-9 and the patients’ performance status are used as prognostic indicators of resectability and survival (6). Current guidelines recommend, if feasible, surgical resection, followed by adjuvant chemotherapy with (modified) FOLFIRINOX (5-fluorouracil, leucovorin, irinotecan, and oxaliplatin) (7) for fit patients or gemcitabine ± capecitabine (8) for frail patients. Several studies report beneficial signals for a perioperative strategy (9), but NCCN guidelines only recommend a neoadjuvant approach within clinical trials or with risk factors present (large primary tumor, large regional lymph nodes, excessive weight loss, and extreme pain) (5). The best regimen for neoadjuvant chemo(radio)therapy is currently not defined. Despite all efforts, recurrence rates after curative intended surgery remain high with relapse rates ranging between 60% and 79% after 3 years (7).

### Borderline Resectable Tumors

A borderline resectable tumor encompasses large arteries with ≤180° circumference and venous contact that allows for complete resection and vein reconstruction (5). Neoadjuvant chemo(radio)therapy is encouraged to attempt downstaging and treat micrometastases. The best regimen to use in this setting is not established. If feasible, neoadjuvant treatment should be followed by surgical resection and adjuvant therapy. Ideally, systemic treatment should comprise a total period of 6 mo including the preoperative regimen (9).

### Locally Advanced Tumors

Indicators of local unresectability include encasement of >180° of the superior mesenteric artery or celiac axis, aortic involvement, or unreconstructible superior mesenteric vein or portal vein (5). These patients (30%–40%) should receive neoadjuvant chemo(radio)therapy. Surgical exploration should be considered if patients respond favorably (10).

### Metastatic Disease

Approximately 40% of patients initially present with disseminated disease. Well-established first-line options in the metastatic setting are gemcitabine (2), gemcitabine + erlotinib (11), gemcitabine + nab-paclitaxel (12), or (modified) FOLFIRINOX (13). The choice of therapy is based on the patients’ age, performance status, and comorbidities (14). Patients with relevant comorbidities [Eastern Cooperative Oncology Group (ECOG) score ≥2] should preferably receive single-agent treatment or best supportive care (BSC). Upon progress under a gemcitabine-based therapy, 5-fluorouracil plus either (nanoliposomal) irinotecan (15) or oxaliplatin (16, 17) can be offered. Despite the lack of randomized trials investigating a subsequent therapy after failure of (m)FOLFIRINOX, second-line gemcitabine ± nab-paclitaxel can be considered (18).

Tailored therapies have demonstrated a benefit for small subgroups of patients, such as poly (ADP-ribose) polymerase (PARP) inhibitors in germline BRCA1/2 (gBRCA) mutations (19) or immune checkpoint inhibitors in mismatch repair (MMR) deficiency or high microsatellite instability (MSI-H) (20). Patients with advanced PDA should undergo molecular profiling early, in particular gBRCA testing, as platinum-based treatments are particularly effective in gBRCA-mutated PDA (21) and the PARP inhibitor olaparib should be considered as maintenance therapy (19).

## GEMCITABINE AS A CORNERSTONE IN PDA THERAPY

Gemcitabine (2′,2′-difluoro-2′-deoxycytidine, dFdC) is a synthetic deoxycytidine nucleoside analog that first demonstrated a survival benefit in PDA in 1997 (2) and remains a cornerstone in current PDA therapies. It is one of the most widely used antineoplastic agents in clinical oncology and a potent drug in several tumor entities, such as breast, ovarian, bladder, or nonsmall lung cancer (22). As compared with other cytotoxic chemotherapies, gemcitabine is generally considered a tolerable compound for the majority of patients with PDA. Treatment-related adverse events include hematological (e.g., neutropenia, anemia, thrombocytopenia, thromboembolism) and nonhematological (e.g., vomiting, fatigue, elevated levels of alanine aminotransferase) side effects. Importantly, these are mostly clinically manageable and fatal treatment-related events have been reported in only a few (<1% to 4%) patients (12, 13).

## GEMCITABINE MECHANISMS OF ACTION

**Figure 2.**
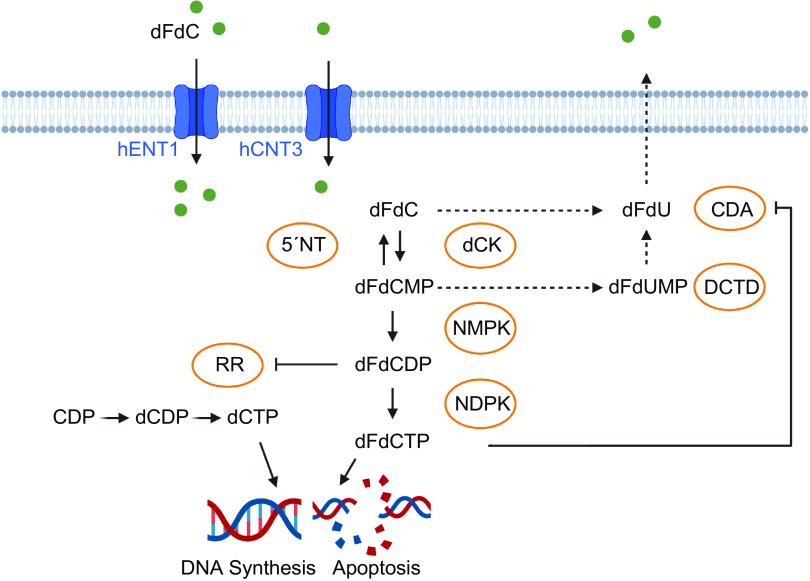
Mechanisms of gemcitabine action. The prodrug dFdC is transported through the cell membrane by nucleoside transporters and phosphorylated to its active diphosphate (dFdCDP) and triphosphate forms (dFdCTP). dFdCTP is incorporated into the DNA, ultimately terminating DNA synthesis. dFdCMP is dephosphorylated to dFdC via 5′nucleotidase, dFdC and dFdCMP are inactivated by CDA and DCTD. As self-potentiation mechanisms, binding of dFdCDP inactivates RR and thereby eliminates competing dCTP pools, and binding of dFdCTP inactivates CDA. CDA, cytidine deaminase; CDP, cytidine-5′-diphosphate; dCDP, 2′-deoxycytidine-5′-diphosphate; dCK, deoxycytidine kinase; DCTD, deoxycytidylate deaminase; dCTP, 2′-deoxycytidine-5′-triphosphate; dFdC, 2′,2′-difluoro-2′-deoxycytidine; dFdCDP, 2′,2′-difluoro-2′-deoxycytidine-5′-diphosphate; dFdCMP, 2′,2′-difluoro-2′-deoxycytidine-5′-monophosphate; dFdCTP, 2′-difluoro-2′-deoxycytidine-5′-triphosphate; dFdU, 2′-deoxy-2′,2′-difluorouridine, dFdUMP, 2′-deoxy-2′,2′-difluorouridine monophosphate; hCNT3, human concentrative nucleoside transporter 3; hENT1, human equilibrative nucleoside transporter 1; NDPK, nucleotide diphosphate kinase; NMPK, nucleotide monophosphate kinase; 5′NT, 5′ nucleotidase; RR, ribonucleotide reductase. Image created with BioRender and published with permission.

To understand the mechanisms of resistance to gemcitabine therapy, it is important to acknowledge its mechanism of action, as outlined in this section and in Fig. 2. As a hydrophilic molecule, gemcitabine requires nucleoside transporters, such as human equilibrative nucleoside transporters (hENTs) and human concentrative nucleoside transporters (hCNTs), to permeate through the cellular lipid membrane. Gemcitabine is transported into cells by hENT1, hENT2, hCNT1, hCNT2, and hCNT3, while most of the uptake is mediated through hENT1 (23, 24). Intracellularly, the prodrug dFdC is metabolized via a three-step phosphorylation. dFdC is phosphorylated by deoxycytidine kinase (dCK) to gemcitabine monophosphate (2′,2′-difluoro-2′-deoxycytidine monophosphate, dFdCMP) and then converted to its active diphosphate (dFdCDP) and triphosphate forms (dFdCTP) (23, 24).

Gemcitabine exhibits its cytotoxic effect primarily through the inhibition of DNA synthesis. dFdCTP competes with deoxycytidine triphosphate (dCTP) to be incorporated into the DNA by DNA polymerase during replication. Once incorporated into the DNA, dFdCTP leads to a premature chain termination after insertion of another nucleotide triphosphate (dNTP). This nonterminal position of dFdCTP, referred to as masked chain termination, inhibits the removal of dFdCTP by DNA repair enzymes, ultimately leading to apoptosis (23, 24). Gemcitabine induces cell cycle arrest particularly in the S phase, but higher concentrations may also cause apoptosis of cells in G1 and G2/M phases (25).

Gemcitabine metabolites can also inhibit other metabolic enzymes to indirectly increase their relative cytotoxicity, a process termed self-potentiation. Here, covalent binding of dFdCDP inactivates ribonucleotide reductase (RR) that catalyzes the conversion of ribonucleotides to deoxynucleotides. By depleting competing dCTP pools that are necessary for DNA synthesis, gemcitabine increases its probability of incorporation into the DNA.

Finally, there are several metabolic reactions that counter the cytotoxic potential of gemcitabine. Gemcitabine (dFdC) is inactivated by cytidine deaminase (CDA) to dFdU (2′-deoxy-2′,2′-difluorouridine) and gemcitabine monophosphate (dFdCMP) is inactivated by deoxycytidylate deaminase (DCTD) to dFdUMP (23, 24). In addition, cytosolic 5′nucleotidase 1A (NC5C1A) dephosphorylates dFdCMP to the prodrug dFdC (26).

## MECHANISMS OF GEMCITABINE RESISTANCE

**Figure 3.**
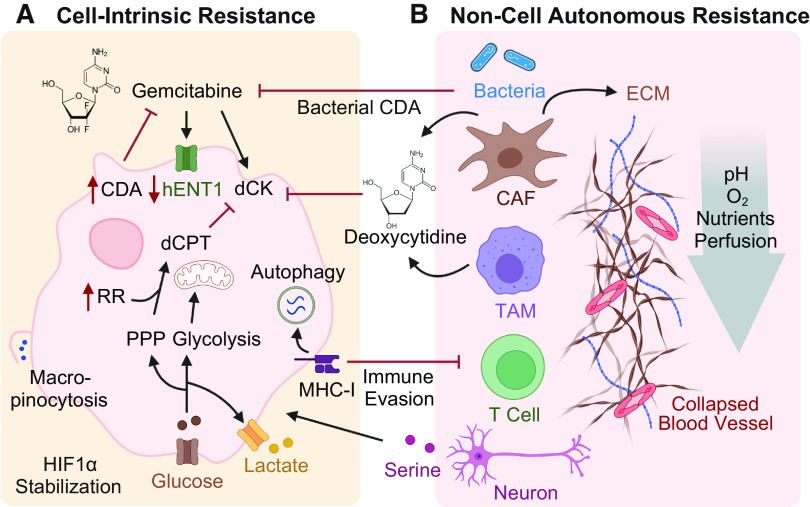
Cell-intrinsic and noncell autonomous gemcitabine resistance mechanisms. *A*: cell-intrinsic mechanisms that render cancer cells less impacted by gemcitabine. Gemcitabine uptake is reduced by downregulation of hENTs; gemcitabine activation is directly inhibited by downregulation of dCK and indirectly by upregulation of RR and glucose uptake, which increases competing nucleic acid pools; gemcitabine detoxification is enhanced by upregulation of CDA. Activation of macropinocytosis and autophagy provide nutrients and promote immune evasion by degrading MHC-I molecules. *B*: resistance mechanisms promoted by the surrounding nonmalignant cells. CAFs and TAMs release deoxycytidine, which outcompetes gemcitabine uptake through dCK. TAMs-induced upregulation of CDA and bacterial CDA detoxify gemcitabine. CAFs produce abundant ECM, which generates high interstitial pressure, vascular collapse, and hypoxia, limiting the delivery of gemcitabine to cancer cells. Red arrows indicate up- or downregulation. CAFs, cancer-associated fibroblasts; CDA, cytidine deaminase; dCK, deoxycytidine kinase; ECM, extracellular matrix; hENT1, human equilibrative nucleoside transporter 1; PPP, pentose phosphate pathway; RR, ribonucleotide reductase; TAMs, tumor-associated macrophages. Image created with BioRender and published with permission.

Therapy resistance refers to the ability of cancer cells to evade the cytotoxic effects of drugs and can be classified as primary or acquired resistance. In primary resistance, the treatment is ineffective from the beginning, whereas, in the acquired form, resistance mechanisms occur after initial responsiveness. Chemoresistance can arise through reprogramming of processes within cancer cells, either during carcinogenesis or in response to treatment. These are both referred to as cell-intrinsic mechanisms. In addition, chemoresistance can be driven by the TME, a more recent concept termed noncell autonomous resistance. Each of these concepts provides unique challenges to effective treatment of PDA, which is discussed here and in Fig. 3.

### Cell-Intrinsic Mechanisms of Chemoresistance

Cancer cells can undergo modifications that facilitate their survival in the presence of cytotoxic agents. These include metabolic mechanisms such as reducing gemcitabine uptake, increasing gemcitabine detoxification, or upregulating endogenous substrates that outcompete gemcitabine activation. Other resistance mechanisms include epigenetic reprogramming or changes in cell state that can also cause cancer cells to be less impacted by antimetabolite therapies.

#### Metabolic resistance mechanisms.

Gemcitabine uptake into the cell is mediated by nucleoside transporters. The existence of a potential association between the expression of nucleoside transporters and gemcitabine resistance remains controversial. Some studies have demonstrated that the absence of hENT1 as assessed by immunohistochemistry in tumor tissue was associated with shortened survival in gemcitabine-treated patients with PDA and have proposed hENT1 expression as a prognostic biomarker for gemcitabine sensitivity (27). In contrast, a prospective randomized phase III trial showed no survival difference regarding tumor tissue expression of hENT1 in PDA patients receiving adjuvant gemcitabine (28).

As gemcitabine is a prodrug, activation is crucial for its effect to unfold. Accordingly, downregulation or inactivation of dCK, which activates gemcitabine by phosphorylation, is a well-acknowledged resistance mechanism (29, 30). Supporting this, high expression levels of dCK in cancer tissue were associated with better survival in patients with PDA receiving adjuvant gemcitabine (31).

For activated gemcitabine to be effective, it must outcompete endogenous deoxycytidine within the cell. Ribonucleotide reductase (RR) is a key enzyme in the de novo nucleotide synthesis pathway. Increased activity of RR amplifies the dNTP pool, subsequently reducing the incorporation of gemcitabine into the DNA through direct molecular competition. Overexpression of the RR subunit M1 (RRM1) has been observed in gemcitabine resistant pancreatic cancer cell lines (32). Indeed, high expression of RRM1 has been associated with lower overall survival (OS) in resected PDA patients receiving adjuvant gemcitabine (33). Increased glucose uptake also contributes to resistance. Besides the bioenergetic roles of the glycolysis pathway, glucose also feeds into anabolic processes. This includes increased flux into the pentose phosphate pathway (PPP) to drive higher de novo synthesis of pyrimidine nucleotides, which attenuates the cytotoxic effect of gemcitabine in a similar fashion (34).

Gemcitabine detoxification is mainly driven by CDA. Interpatient variability in CDA activity is caused by genetic polymorphisms of the CDA gene. A small pilot study showed, that “rapid deaminator” status, or high CDA activity, was associated with poor clinical outcome in patients with PDA receiving gemcitabine-based treatment (35). Conversely, low CDA activity in the serum was associated with gemcitabine toxicity in patients, suggesting CDA functional status as a marker to identify patients at risk for developing severe toxicities with gemcitabine treatment (36). Intriguingly, in a PDA mouse model, nab-paclitaxel, the compound most frequently combined with gemcitabine, has been shown to potentiate gemcitabine activity by driving the degradation of CDA through the induction of reactive oxygen species (37).

New insights are still being made into the basic metabolism related to gemcitabine processing. Recently, NT5C1A has been reported to contribute to gemcitabine inactivation by reversing the initial phosphorylation step of dFdC to dFdCMP. Consequently, overexpression of NT5C1A was found to confer gemcitabine resistance in PDA tumor cells and in tumor-bearing mice (26).

Other rewired metabolic pathways have been noted to correlate with gemcitabine resistance. For example, PDA cells have been found to increase the expression of fatty acid synthase (FASN) expression during disease progression. This increased FASN expression also correlates with poor response to gemcitabine (38). Furthermore, pancreatic cancer cells combat nutrient limitations by activating scavenging and recycling pathways. As such, macropinocytosis, an endocytic process that internalizes extracellular fluids and macromolecules into cells, can promote resistance to chemotherapy (39, 40). Autophagy, a regulated process in which cells consume their own proteins and organelles into the lysosome, has been shown to be constitutively elevated in PDA (41). Autophagy not only provides an internal source of nutrients for energy but also promotes immune evasion by selectively degrading major histocompatibility complex class I (MHC-I) molecules (41).

#### Epithelial-mesenchymal transition and acquisition of stemness.

Epithelial-mesenchymal transition (EMT) is a biological process in which epithelial cells acquire a mesenchymal phenotype with enhanced invasive properties (42). EMT plays an important role in embryonic development, wound healing, and tumorigenesis. In cancer, EMT is linked to increased cell motility and the acquisition of stem cell-like features, which is pivotal for tumor dissemination. At metastatic sites, invading cancer cells undergo mesenchymal to epithelial transition (MET), the reverse of EMT, to proliferate at distant organs (43). EMT is canonically regulated by various transcription factors, including Snail, Twist, and zinc-finger E-box binding homeobox 1 (Zeb) and controlled by key signaling pathways, such as ERK, Notch, and NF-κB (42).

EMT has been linked to therapy resistance through several mechanisms. Reversal of EMT via silencing of Zeb-1 has been shown to correlate with the expression of epithelial markers and restored drug sensitivity in resistant cell lines (44). In a mouse model of PDA, deletion of the key transcription factors Snail or Twist led to an increased expression of nucleoside transporters in tumors and enhanced sensitivity to gemcitabine treatment (45). Gemcitabine treatment has also been found to induce the expression of Midkine (MK), a heparin-binding growth factor. MK can activate Notch2 signaling to drive both EMT and chemoresistance in PDA cells (46).

Cancer cells that undergo EMT can acquire stem cell-like properties and show an increase in CD44 expression, which is frequently used as a molecular marker for cancer stem cells (CSCs) (47). CSCs are defined as a small subpopulation of inherently resistant cancer cells with self-renewal and differentiation properties. This allows CSCs to survive treatment, enrich for populations resistant to therapy, and serve as a reservoir to promote tumor relapse (48). CD44, a non-kinase transmembrane receptor that binds to hyaluronan, exists in alternatively spliced variants that have been shown to regulate EMT and epithelial plasticity. Gemcitabine treatment can drive both upregulation of CD44 expression and CD44 isoform switching through insulin-like growth factor receptor 1 to confer gemcitabine resistance in PDA cells (49). Beyond CD44, the self-renewal transcription factor SOX2 is commonly associated with CSCs. Here, SOX2 can contribute to cancer proliferation, EMT, and resistance to therapy (50). As an example, the GLI-SOX2 signaling axis has been implicated in the formation of tumor-initiating cells, a term often associated with CSCs, and gemcitabine resistance in PDA (51).

These are just a few examples of the complex interplay between EMT, stemness, cell states, oncogenic signaling, and responses to chemotherapy that occur within cells. A more refined understanding of these interactions promises to reveal new pathways to target chemoresistance in PDA. However, changes to each of these processes also drive compensatory changes in the tumor milieu beyond the cancer cells themselves.

### Noncell Autonomous Mechanisms of Chemoresistance

The pancreatic TME, which accounts for ∼90% of the tumor composition, is composed of an abundant extracellular matrix (ECM), nonmalignant cells (e.g., fibroblasts, adipocytes, endothelial cells, immune cells), and acellular components such as growth factors and cytokines (52). Cancer cells are dwarfed by the noncancer cell populations in this vast and dense TME. During tumorigenesis, emerging neoplastic lesions co-opt surrounding normal support cells in tissues to acquire nutrients and growth factors that support their proliferation (53). The different cell populations dynamically exchange signaling molecules and metabolites that promote cancer cell proliferation and mediate immune suppression (54). Importantly, several of these cross talk mechanisms directly mediate chemoresistance (55–61).

#### Cancer-associated fibroblasts.

Fibroblasts in the normal pancreas were originally described as pancreatic stellate cells (PSCs) that acquire distinct features upon transition to cancer-associated fibroblasts (CAFs), although recent evidence suggests this is a more complex and poorly understood process (62). CAFs constitute the major cellular component of the TME, and increased focus on these populations in recent years has revealed a previously unappreciated diversity in subsets with unique biological functions (63). Efforts are being made to define subclasses of CAFs such as myofibroblastic CAFs (myCAFs), inflammatory CAFs (iCAFs), and antigen-presenting CAFs (apCAFs), each with distinct behaviors in shaping the microenvironment (64, 65).

Accumulating evidence suggests that CAFs act to promote resistance to gemcitabine through several mechanisms. CAFs produce abundant ECM including collagen and the nonsulfated glycosaminoglycan hyaluronan. The ECM generates a high interstitial pressure, promoting vascular collapse and hypoperfusion, which limits the delivery of therapeutics to neoplastic cells (66). Interestingly, a high proportion of stroma is associated with a poor OS independently of tumor stage or cancer entity (66). In PDA, desmoplastic stroma and high deposition of hyaluronan confer poor patient survival (67). Conversely, a histological signature of increased tumor-infiltrating leucocytes as an indication of the host immune response is associated with prolonged progression-free survival (PFS) in resected patients with PDA (68).

The impaired vasculature within PDA tumors further restricts oxygen diffusion, leading to regional hypoxia. Areas of hypoxic tumor tissues are known to be more resistant to therapy (69). Within these regions, low oxygen levels stabilize hypoxia-induced factors (HIFs), which promote EMT and are frequently overexpressed in gemcitabine resistant PDA cells (70). HIF-1α has also been shown to be stabilized by MUC1 signaling, which drives the rewiring of anabolic glucose metabolic networks in PDA cells (34). Cancer cells undergo anerobic glycolysis even in the presence of oxygen and produce high levels of lactate. Increased glycolytic flux drives glucose intermediates into the PPP, which increases the biosynthesis of nucleotides that outcompete gemcitabine (34).

Apart from creating a physical barrier and hypoperfusion resulting in hypoxia, CAFs contribute to direct and indirect chemoresistance mechanisms. Triggered by cancer cells, CAFs are promoted to synthesize and release glutamine, which can be consumed by cancer cells to promote their proliferation (71). Furthermore, CAFs have been shown to secrete deoxycytidine, which directly competes with gemcitabine, promoting drug resistance (55). Indirectly, CAFs have been shown to be a source of the matricellular protein cysteine-rich angiogenic inducer 61 (CYR61), which downregulates the expression of the nucleoside transporters hENT1 and hCNT3, thereby reducing gemcitabine uptake (56). Other work has demonstrated that myCAFs and TAMs promote chemoresistance by secreting insulin-like growth factors (IGF) 1 and 2, which support PDA cell survival via activation of the insulin/IGF receptor survival signaling pathway (57).

Overall, CAFs have been found to play an important role in numerous protumorigenic functions. Unfortunately, direct targeting of bulk CAF populations has not provided a straightforward approach to enhance responses to gemcitabine therapy. However, significant optimism remains that our increased understanding of CAF subpopulations might better inform strategies to target the stroma in the future.

#### Tumor-associated macrophages.

PDA tumors are highly inflamed, and macrophages are the most abundant immune cells in the pancreatic TME (72). Macrophage infiltration is thought to be an early event in pancreatic tumorigenesis. As an example, oncogenic KRAS signaling in pancreatic acinar cells during acinar-to-ductal metaplasia can drive the expression of intercellular adhesion molecule-1 (ICAM-1), which acts as a chemoattractant for macrophages (73). Macrophages are derived from a mixed population of tissue-resident cells and circulating monocytes that are converted into tumor-associated macrophages (TAMs) by cancer cells (72, 74). Chemokines, small-molecule proteins that exert their functions by binding to G protein-coupled chemokine receptors, regulate immune cell infiltration, affect tumor immunity, and influence cancer progression (54). CCL2, also known as monocyte chemoattractant protein-1, and CCL5, also referred to as RANTES (regulated on activation, normal T cell expressed, and secreted), are key molecules in macrophage chemotaxis and activation that have been reported to promote tumor proliferation (54).

Depending on their polarization status, macrophages can exert contrasting roles in tumorigenesis and antitumor response (74). Pancreatic TAMs are observed to be in an anti-inflammatory polarization state, largely characterized by their expression of Arginase 1 (Arg1) (75). Proline, produced downstream of arginine catabolism by Arg1, is a critical component of collagen biosynthesis and likely a key component of the establishment and maintenance of the desmoplastic response in PDA. Both protein and metabolite factors derived from PDA cells contribute to the anti-inflammatory polarization of pancreatic TAMs (76), exemplifying the complexity of the dynamic and reciprocal interactions between immune-epithelial cell populations.

In addition, TAMs and other anti-inflammatory macrophages engage in robust nucleotide biosynthesis, driving the release of numerous pyrimidine species. These include deoxycytidine, which inhibits gemcitabine through molecular competition at the level of drug uptake through the dCK (58). Furthermore, TAMs have been shown to induce an upregulation of CDA within PDA cells. This allows cancer cells to rapidly inactivate gemcitabine to dFdU, thereby lowering intercellular gemcitabine concentrations (59).

#### Other cell types in the TME.

An altered gut and pancreas microbiome has been reported as a common feature of PDA. Interestingly, the composition of the microbiome has been closely linked to oncogenesis and PDA patient outcome (77, 78). Beyond regulating host immune response, intratumoral bacteria have been directly linked to chemoresistance. Microbes, primarily gammaproteobacteria, can inactivate gemcitabine through a long isoform of the bacterial CDA, which metabolizes gemcitabine to its inactive form in the tumor (61). This bacteria-induced gemcitabine resistance was abrogated by coadministration of the antibiotic ciprofloxacin in a colon cancer mouse model (61). Numerous clinical studies are underway that aim at modulating the microbiome by the use of antibiotics, dietary changes, or fecal microbiota transplant to augment treatment efficacy and patient outcome (e.g., NCT05462496, NCT04631445, NCT04975217).

Neuronal remodeling, perineural invasion, and neuropathic pain have been consistently associated with PDA and shown to correlate with patient survival (79). Recently, axonal-cancer metabolic cross talk was reported to support PDA growth in nutrient-poor environments. PDA cells may promote neuronal innervation via secretion of nerve growth factor (NGF) to leverage neurons for the supply of neurotrophins, neurotransmitters, or amino acids (60, 80). As an example, peripheral axons have been shown to release serine, a conditionally essential amino acid, that can function to support tumor growth (60). Murine PDA studies have demonstrated therapeutic efficacy of targeting nerve innervation through ablation of sensory nerves (81), anti-NGF therapy (82), or inhibition of the NGF receptor tropomyosin-related kinase (TRK) (80). The TRK inhibitors entrectinib and larotrectinib have been approved in solid tumors with TRK fusion (83, 84); although mainly targeting TRK receptor signaling, future studies should also evaluate whether they inhibit nerve innervation.

Obesity is an established risk factor for PDA. Nevertheless, the role of adipocytes and lipid metabolism is often overlooked. Emerging evidence indicates that cancer-associated adipocytes promote tumor growth and drug resistance through complex mechanisms, including the release of tumor-promoting growth factors and adipocytokines. Obesity-induced microbiota dysbiosis as well as reduced blood flow, hypoxia, and inflammation in adipose tissue may further contribute to resistance (85).

Finally, the association between PDA and increased risk for venous thromboembolism suggests a close interplay between cancer cells and platelets. Indeed, activated platelets promote metastasis and drive gemcitabine resistance. Platelet-derived nucleotides including ADP and ATP upregulate the expression of the EMT transcription factor Slug, which can both increase expression of CDA and decrease hENT1 levels, collectively diminishing the effectiveness of gemcitabine (86). A phase III clinical trial evaluating the addition of the ADPP2Y12 inhibitor clopidogrel to chemotherapy in PDA terminated due to recruitment difficulties (NCT02404363).

## POTENTIAL APPROACHES TO OVERCOME GEMCITABINE RESISTANCE

Given the importance of gemcitabine in PDA treatment, extensive efforts have focused on exploring methods to circumvent resistance mechanisms. The following section will highlight some therapeutic avenues that have been attempted to resensitize resistant PDA cells to gemcitabine-centered treatments. Although many early efforts have failed to achieve clinical efficacy, the lessons learned hold promise for a much-needed breakthrough.

### Enhancing Pharmacokinetic Properties of Gemcitabine

In an effort to enhance the efficacy of gemcitabine through improved biostability and bioavailability, a large number of gemcitabine prodrugs and chemical modifications have been developed.

PEGylation, the conjugation of poly(ethylene glycol) (PEG) to a biological compound, has proven useful in the treatment of several diseases, as it improves drug stability, prolongs circulation time, decreases immunogenicity, and reduces metabolic degradation (87). Indeed, PEGylated gemcitabine increased the bioavailability and prolonged the circulation time in plasma in animals and enhanced cytotoxicity in human PDA cell lines (88).

Another efficient way to improve drug delivery of gemcitabine is through covalent coupling of squalene, a natural lipid, to the 4-NH_2_ group. Gemcitabine-squalene can passively diffuse into cancer cells without relying on nucleoside transporters and demonstrated superior effectiveness compared with gemcitabine in vitro and in vivo (89). Further attempts to enhance cellular uptake include derivatives containing a fatty acid side chain. CP-4126 is an oral formulation of gemcitabine 5′ elaidic acid ester, which can traverse cell membranes by passive diffusion and was found to be protected from deaminase deactivation. Unfortunately, a phase I trial was terminated due to poor absorption, rapid presystemic metabolism, and consequently the high amount of capsules the patients were required to take (90).

To bypass the rate-limiting step of primary phosphorylation of gemcitabine through the dCK, a gemcitabine phosphoramidate prodrug was developed that was more active in dCK-deficient cell lines (91). Similarly, NUC-1031, a gemcitabine phosphoramidate prodrug, has the ability to enter the cell independent of nucleoside transporters due to its increased hydrophobicity. As a modified dFdCMP compound, it also bypasses the rate-limiting step of dCK-mediated phosphorylation and avoids CDA-mediated catabolism. Both preclinical and early clinical data indicated positive PDA tumor responses to NUC-1031 in the context of gemcitabine resistance (92), unfortunately, the corresponding phase III trial (NCT03610100) was suspended due to futility.

These are a few examples of the numerous gemcitabine modifications that have been explored as an approach to combat resistance by altering its metabolism, however, challenges such as absorption, site-specific delivery, and release of the drug remain.

### Targeting Fibroblasts to Remodel Stroma

The sonic hedgehog (Shh) signaling pathway is a key pathway in pancreas development. Deregulation of hedgehog signaling is known to promote carcinogenesis and contribute to desmoplasia formation (93). Consequently, inhibition of the Shh signaling pathway was investigated as a stroma-targeting therapeutic strategy in PDA. The coadministration of IPI-926, an inhibitor of the Shh pathway, increased intratumoral vascular density and intratumoral gemcitabine concentration in murine PDA models (94). However, attempts to translate these findings into a clinical benefit were largely unsuccessful, leading to a worse prognosis forcing the clinical trial to close early (95). Mechanistic follow-up studies have pointed toward a tumor-restraining role for the PDA stroma, as fibroblast depletion has been found to promote metastasis.

Additional approaches to target the stromal fibroblasts have yielded more promising results. Hyperactivated focal adhesion kinase (FAK) in cancer cells has been found to be an important regulator of TME in preclinical PDA mouse models (96). The dual FAK1 and FAK2 inhibitor, VS-4718, reduced tumor progression and fibrosis in these models. Importantly, VS-4718 in combination with gemcitabine or nab-paclitaxel led to longer survival (96, 97), however, the subsequent phase I study evaluating VS-4718 in combination with gemcitabine and nab-paclitaxel was terminated by the company’s decision to deprioritize VS-4718 (NCT02651727). Nevertheless, these results suggest that stromal targeting still has potential in PDA.

### Targeting the Matrix to Enhance Drug Delivery

As remodeling the TME by targeting fibroblasts to increase chemotherapy access has yielded mixed results, several attempts have been made to directly target the ECM. Hyaluronan is a predominant ECM component that retains water and is responsible for elevated interstitial pressure in PDA tumors (98). Accordingly, enzymatic depletion of hyaluronan by human recombinant PH20 hyaluronidase (PEGPH20) was pursued as a potential therapeutic strategy aiming to lower interstitial pressure and subsequently increase chemotherapy diffusion, while leaving the tumor-restraining fibroblasts intact. Preclinical studies in genetically engineered mouse models of PDA with PEGPH20 induced re-expansion of blood vessels, increased the intratumoral delivery of chemotherapeutics, and led to effective inhibition of tumor growth in vivo (99). However, subsequent clinical phase I/II trials investigating PEGPH20 in combination with chemotherapy provided inconsistent results, prompting further investigation. Disappointingly, a follow-up phase III trial demonstrated that the addition of PEGPH20 to gemcitabine/nab-paclitaxel did not improve PFS or OS as compared to chemotherapy alone (100).

To target the particularly resistant hypoxic areas of the tumor, Evofosfamide (TH-302), a prodrug of the DNA alkylator bromo-isophosphoramide mustard that is preferentially activated under hypoxic conditions, was designed. A phase II and subsequent phase III study of evofosfamid and gemcitabine significantly improved PFS, but not OS, in PDA (101, 102).

Accordingly, improvements are needed to further increase the efficacy of these approaches.

### Targeting Myeloid Cells to Reduce Chemoresistance

Given their ability to promote chemoresistance through multiple mechanisms, TAMs have become an attractive target to combat resistance. Inhibition of macrophage trafficking by the colony-stimulating factor 1 receptor (CSF1R) inhibitor AZD7507 in combination with gemcitabine increased the survival in a murine PDA model (58). A phase I study of anti-CSF1R in combination with an anti-PD-L1 monoclonal antibody in patients with advanced colorectal or pancreatic cancer was recently completed with no unexpected toxicities (NCT02777710) (103). Similarly, gemcitabine combined with pharmacological depletion of TAMs has provided a synergistic antitumor effect in PDA mouse models (104). In addition, the CSF1R antagonist GW2580 augmented the cytotoxic effect of gemcitabine in a gemcitabine resistant PDA mouse model (59). Collectively, these studies demonstrate significant promise for targeting myeloid cells in combination with chemotherapy.

Targeting macrophage chemotaxis by impairing CCL2/CCR2 or CCL5/CCR5 chemokine-chemokine receptor signaling axis represents another promising therapeutic approach. Inhibition of CCR2 or dual CCR2/CCR5 antagonists in combination with chemo- or immunotherapy is currently being tested in clinical trials (e.g., NCT02345408, NCT03496662, NCT03184870, NCT03767582). Analysis of the data generated in these trials will be equally intriguing in both the patient response to therapy, shifts in immune landscape, and potential reprogramming of the TME to these interventions.

### Targeting Metabolic Reprogramming

PDA cells require dramatically reprogrammed metabolism to survive within the nutrient-deprived pancreatic TME (105). Thus, metabolic alterations represent an attractive therapeutic target that holds promise to reverse gemcitabine resistance. For example, increased de novo nucleotide synthesis has been highlighted as a cell-intrinsic gemcitabine resistance mechanism. Accordingly, inhibition of dihydroorotate dehydrogenase (DHODH), a key enzyme in the pyrimidine biosynthesis pathway, has the ability to reverse chemoresistance (34). Currently addressing this, a phase IA/IB clinical trial aims to investigate leflunomide in patients with PTEN-null advanced solid malignancies (NCT04997993), although this inclusion criterion is unlikely to capture many patients with PDA.

Anabolic glucose metabolic rewiring has been implicated in the acquisition of gemcitabine resistance, and lactate dehydrogenase inhibitors (LDH) have shown potential to counteract this reprogramming in patient-derived xenografts (106). Interestingly, FX11 administration inhibited pyruvate-to-lactate conversion only in mutant TP53 tumors, highlighting TP53 mutational status as a potential biomarker to predict sensitivity to LDH inhibition. The basis for the reduced LDH-A inhibition in TP53 wild-type cells could be caused by a higher expression of TIGAR (TP53-induced glycolysis and apoptosis regulator), a TP53-inducible protein that decreases levels of fructose-2-6-biphosphate, which suppresses glycolysis by diverting glucose-6-phosphate into the PPP (106). TIGAR expression levels have also been shown to decrease during PDA progression, corresponding to elevated ROS levels and leading to increased migratory and metastatic potential (107). Importantly, this is consistent with the fact that advanced PDA lesions are often associated with loss of p53 function (108) and further links EMT phenotypes (e.g., migration and invasion) to metabolic state and gemcitabine resistance in PDA.

Another interesting approach to combat resistance is targeting gemcitabine detoxification. Accordingly, a recent study demonstrated that the combination of the CDA inhibitor zebularine increased cytotoxicity of gemcitabine in PDA cell lines (109). A pilot clinical trial investigated the addition of the CDA inhibitor tetrahydrouridine (THU) to the pyrimidine nucleoside analog decitabine in patients with advanced chemorefractory PDA (110). This did not yield a clinical benefit, however, it is speculated that the CDA inhibitor dose was too low (110).

The mevalonate pathway that uses acetyl-CoA to produce sterols and isoprenoid metabolites is known to be deregulated in cancer to support tumorigenesis. Statins are widely prescribed as lipid-lowering drugs that inhibit HMG-CoA (3-hydroxy-3-methylglutaryl coenzyme A), the rate-limiting enzyme of the mevalonate pathway (111). Increasing evidence suggests anticancer properties for statins through the depletion of cholesterol and mevalonate pathway intermediates that interfere with posttranslational prenylation and activation of small GTPases (such as Ras, Rho, or Rac) and their downstream signaling (111). Numerous studies point to a plethora of anticancer effects of statins. For example, statins can drive depletion of the mitochondrial electron carrier coenzyme Q, causing severe oxidative stress. Combining mitogen-activated protein kinase (MEK) inhibitors with statins to counteract upregulated compensatory antioxidant metabolic pathways of cancer cells enhanced tumor apoptosis in vivo (112). However, numerous epidemiological and clinical studies that investigated the potential benefit of statins in combination with chemotherapy have yielded inconsistent results. A phase II trial reported no clinical benefit for the addition of low-dose simvastatin to gemcitabine in advanced PDA (113), but repurposing statins for cancer treatment still remains an active area of research with several clinical trials investigating different drug combinations with statins in PDA (e.g., NCT04862260, NCT04245644, NCT03889795).

Type 2 diabetes mellitus (T2DM) is a multifactorial disease involving genetic and environmental factors. T2DM is characterized by hyperglycemia, insulin resistance, and β-cell dysfunction, eventually causing micro- and macrovascular complications. T2DM is associated with an increased risk of developing PDA and unfavorable outcomes (114). Insulin resistance, compensatory hyperinsulinemia, increased levels of insulin-like growth factors (IGFs), and inflammation are hypothesized to contribute to the development and progression of PDA (114). Metformin, the most commonly prescribed antidiabetic drug, lowers blood sugar levels mainly through the suppression of hepatic gluconeogenesis. Preclinical evidence shows that metformin inhibits tumor growth in PDA and many epidemiological studies, although not entirely consistent, suggest a beneficial preventive and therapeutic effect of metformin in patients with underlying T2DM (114). A potential therapeutic benefit for metformin in PDA is currently being investigated in multiple clinical trials (e.g., NCT02336087, NCT02048384, NCT04245644, NCT02978547).

Initially, it was thought that inhibition of key metabolic pathways would prove more difficult for cancer cells to circumvent than targeting signaling pathway inhibition. However, cancer metabolism appears more plastic than originally anticipated. For example, PDA cells can dynamically reprogram glutamine metabolism to bypass glutaminase inhibition (115). However, preclinical studies have shown promising activity for less specific pan-glutaminase inhibitors (116) and for a combination of glutamine inhibitors with chemotherapy (117). Recently completed and ongoing basket trials investigating the glutaminase inhibitor telaglenastat (CB-839) in patients with solid tumors harboring specific mutations (e.g., NCT03872427, NCT02071862, NCT03965845) will provide better insight for this in humans.

## SUMMARY

Despite numerous research efforts, conventional chemotherapy containing gemcitabine remains a major component of the standard-of-care (neo)adjuvant and palliative treatment in PDA. Chemoresistance poses a major challenge in PDA therapies. Apart from cell-intrinsic mechanisms, the pancreatic TME promotes proliferation and plays a key role in gemcitabine resistance. Most strategies targeting gemcitabine resistance have not yielded satisfactory results due to upregulation of alternative pathways, metabolic rewiring, and a lack of selective inhibitors. A better understanding of the multifaceted resistance mechanisms, only covered in brief in this review, has the ability to provide numerous avenues that may increase clinical responses and improve survival in this deadly disease.

## GRANTS

A.K.B is supported by a fellowship through the German Cancer Aid Foundation (Mildred-Scheel-Postdoktorandenstipendium). C.J.H. is supported by a National Pancreas Foundation Research Grant, a Sky Foundation Research Grant, a V Scholar Award (V2021-026), a UCI Anti-Cancer Challenge Pilot Award, a UC Pancreatic Cancer Consortium Pilot Award, R00CA241357, and the Chao Family Comprehensive Cancer Center Support Grant P30CA062203.

## DISCLOSURES

No conflicts of interest, financial or otherwise, are declared by the authors.

## AUTHOR CONTRIBUTIONS

A.K.B. prepared figures; A.K.B. drafted manuscript; A.K.B. and C.J.H. edited and revised manuscript; C.J.H. approved final version of manuscript.
